# Assessment of Eating Disorders and Eating Behavior to Improve Treatment Outcomes in Women with Polycystic Ovary Syndrome

**DOI:** 10.3390/life12111906

**Published:** 2022-11-16

**Authors:** Tea Shehu Kolnikaj, Rok Herman, Andrej Janež, Mojca Jensterle

**Affiliations:** 1 Department of Endocrinology, Diabetes and Metabolic Diseases, University of Medicine Tirana, 1000 Tirana, Albania; 2 Department of Endocrinology, Diabetes and Metabolic Diseases, University Medical Center Ljubljana, 1000 Ljubljana, Slovenia; 3 Department of Internal Medicine, Faculty of Medicine, University of Ljubljana, 1000 Ljubljana, Slovenia

**Keywords:** polycystic ovary syndrome, feeding and eating disorders, eating behavior, obesity, anti-obesity drugs

## Abstract

The essential role of the frequent coexistence of mental disorders and polycystic ovary syndrome (PCOS) is being increasingly recognized in the management of PCOS patients since it influences the success of weight loss interventions. Patients frequently experience disrupted eating behaviors, evidenced by the high prevalence of eating disorders in this population. Therefore, assessment and potential modification of eating disorders and eating-related behavior might be especially relevant to improve obesity treatment outcomes in this population, which remains the most efficient causal treatment in PCOS patients with high metabolic risk. Following a literature overview on common eating disorders and eating behaviors in PCOS, the aim of this review was to explore the prevalence and underlying mechanisms behind those occurrences. Understanding the clinical relevance of those associations and the addition of the assessments of eating disorders as well as eating phenotypes, eating chronotypes, and eating content as essential determinants of eating behavior could aid in the successful management of women with PCOS. In addition, the review also covers the potential of using eating disorders and eating behavior as a tool for the personalization of obesity treatment in PCOS.

## 1. Introduction

Polycystic ovary syndrome (PCOS) is a heterogeneous, multifactorial disease that leads to menstrual and ovulatory irregularities, infertility, hyperandrogenism, and metabolic disturbances [1]. It is recognized as the most prevalent endocrine/metabolic disorder in reproductive-aged women [2]. Despite continuous research efforts, the unifying pathophysiological mechanisms that could explain the etiology of this complex disorder are still not fully understood. The interplay between genetic and epigenetic factors, mitochondrial dysfunction, altered protein and miRNA profiles, and environmental factors that also contribute to obesity [3] lead to exaggerated gonadotropin-releasing hormone (GnRH) pulsatility with hypersecretion of luteinizing hormone (LH) and insulin resistance (IR). Compensatory hyperinsulinemia adds to increased ovarian androgen production and impaired oocyte development [4].

Obesity and PCOS are strongly interrelated with multiple common underlying mechanisms. Obesity exacerbates all hormonal and clinical features of PCOS, and women with PCOS appear at a higher risk of developing metabolic syndrome and obesity [5]. Weight gain contributes to the severity of IR in PCOS and leads to over-stimulation of the post-receptor mitogen-activated protein kinase (MAP-K) insulin pathway with consequent implications on steroidogenesis and ovarian function [6]. PCOS has been associated with many established co-morbidities related to obesity, including type 2 diabetes, cardiovascular disease, infertility, and mental disorders such as anxiety, depression, and eating disorders (EDs) [7,8,9,10].

The essential role of the frequent coexistence of mental disorders and PCOS is being increasingly recognized in the management of PCOS. Almost all women diagnosed with PCOS are significantly affected by various psychological disorders in all dimensions of their life compared to the general population [11]. Their quality of life is impaired by psychosocial concerns related to PCOS. They experience body dissatisfaction, sexual and relational dysfunction, femininity and fertility concerns, loss of self-confidence, and often psychiatric disorders such as mood disorders, major depressive and bipolar disorder, anxiety, EDs, and borderline personality disorder [12]. Weight reduction is the main treatment target in PCOS women who are overweight or obese. Even a minor weight loss of 5–10% can significantly improve fertility and metabolic and psychological symptoms in this population [13]. Unfortunately, this goal is often unattainable because various barriers prevent weight loss and maintenance. Therefore, assessment and potential modification of EDs and eating-related behavior might be especially relevant to improve the outcomes of weight loss interventions in this population.

Assessment and potential modification of EDs and eating-related behavior have been shown to be relevant to improve the outcomes of weight loss interventions in the general population. With weight management being one of the primary treatment targets in the management of women with PCOS, the same principles and focus should be placed on those aspects of the disorder in order to improve metabolic and reproductive outcomes. Following a literature overview on common EDs and eating behaviors in PCOS, the aim of this review was to explore the prevalence and underlying mechanisms behind those occurrences and their implications for the management of women with PCOS. We also considered how the assessment of EDs and eating behaviors could aid in the personalization of obesity treatment in PCOS.

## 2. Eating Disorders in PCOS

### 2.1. Classification of Eating Disorders

EDs are serious conditions characterized by persistent eating behaviors that negatively impact physical health and disrupt psychosocial functioning [14]. The most common EDs are anorexia nervosa, bulimia nervosa, and binge eating disorder [15]. Based on the DSM-5 classification of mental disorders, there is also a category of other specified feeding or eating disorders (OSFED) that do not meet the full criteria for the diagnosis of major disorders and include atypical anorexia nervosa, bulimia nervosa, and binge eating disorder of low frequency and/or limited duration, purging disorder, and night eating disorder.

### 2.2. Prevalence of Eating Disorders in PCOS

PCOS is frequently related to an extreme manifestation of disrupted eating behavior, which is highly prevalent in this population [16,17]. Table 1 summarizes relevant studies to date that have demonstrated the prevalence of various EDs in women with PCOS, and some of them compared the rates to healthy controls.

Most of the studies mentioned in the Table 1 found higher rates of EDs in the PCOS group compared to the control group. The only study that did not demonstrate any differences in the study by Lee et al. [10]. The authors explained that the statistical power of the study was too low to detect these differences. In addition, due to the cross-sectional design, it was difficult to assess the possible confounding role of depression and anxiety on the prevalence of EDs, suggesting that these psychiatric co-morbidities may have a causal role for EDs in the general population. Given that confounding factors in this population may have affected outcomes, replication of this study in other populations, including community cohorts, is suggested by the authors. Furthermore, there is a lack of unifying questionnaires for the diagnosis of EDs in all studies. That could be a reason for the variation in the reported prevalence and possibly the potential overreporting of EDs in some studies [21].

Further perspective can be gained from a systematic review and meta-analysis conducted at Mayo Clinic by Thannickal et al. that included 36 studies. They analyzed different EDs and their prevalence in women with PCOS. No significant association between PCOS and anorexia nervosa (three studies) was found, but a significant association was shown between PCOS and bulimia nervosa (five studies), binge eating disorder (four studies), and other not specified eating disorders (four studies) [23].

Morgan et al. studied EDs in 80 women with facial hirsutism. The prevalence of EDs in this population was 36.3% (22.5% eating disorder not otherwise specified (EDNOS), 12.6% bulimia nervosa, and 1.3% anorexia nervosa), and depression, anxiety, low self-esteem, and poor social adjustment were more common in participants suffering from EDs. There was universal co-morbidity of PCOS in women with hirsutism and EDs, proving again the high prevalence of EDs in the PCOS population [24]. This study performed in 2008 used DSM-IV criteria for the categorization of EDs, where binge eating disorder was not recognized as a disorder and is not mentioned separately in this study [25].

The prevalence of EDs in lean PCOS phenotype is less frequently studied. Jeanes et al. used online surveys, including the Bulimia Investigatory Test, Edinburgh, Food Cravings-Trait 26 Questionnaire, and the Three Factor Eating Questionnaire revised−18 to compare EDs in 45 lean women with PCOS and 40 lean women in the control group. A significantly higher mean binge eating score was shown in lean women with PCOS compared with lean, healthy women (10.9 ± 7.8 versus 7.4 ± 6.0, *p* = 0.024). Lean women with PCOS had a significantly higher proportion of subclinical disordered eating (36%) compared with lean, healthy women (12%) [20].

The existence of these relationships among physiological and psychological factors strongly suggests that medical management of PCOS would greatly benefit from the inclusion of psychological and behavioral approaches [26]. Different authors have already recommended screening this population for depression and eating disorders [21,27,28].

### 2.3. The Casual Link between Eating Disorders and PCOS

Available literature suggests a causal relationship and significant interplay between physiological and psychological factors affecting women with PCOS [26]. Hyperandrogenism, reproductive and metabolic disturbances, and the high prevalence of obesity contribute to body dissatisfaction as well as psychological aspects such as depression, anxiety, and EDs [29].

Hyperandrogenism can alter monoamine balance in the central nervous system and has already been linked to depression [30]. In addition, it stimulates appetite and increases anxiety which may confer risk for binge eating behavior [31,32]. Indirect evidence for the role of hyperandrogenism in EDs is implied by the observation that an antiandrogen receptor antagonist, flutamide, can lead to a substantial decrease in bulimic symptoms [33].

Moreover, metabolic disbalances, especially hyperinsulinemia, contribute to binge eating behavior. High insulin levels, commonly seen in PCOS, lead to postprandial low blood glucose, another potent appetite stimulant primarily in cravings for carbohydrates. Altered insulin levels contribute to weight gain, with increased distress regarding weight status and a common weight cycling phenomenon, often referred to as Yoyo dieting [34]. Once the obese phenotype is fully developed, there is a high equilibrium body weight set-point and numerous mechanisms preventing weight loss, all causing the maintenance of weight loss following lifestyle or pharmacotherapy intervention challenging. Furthermore, hyperandrogenism and hyperinsulinemia can propagate each other in a vicious cycle [35].

Both mechanisms, through the consequential weight gain, inflammation, and clinical symptoms of hyperandrogenism, can lead to body dissatisfaction with impaired self-appearance, which can also be an additional trigger for EDs. Despite the fact that the body dissatisfaction phenomenon is not limited to PCOS but is frequently observed in overweight women in general, body dissatisfaction is more prominent in PCOS women and can lead to EDs. In a study by Karacan et al., 42 women with PCOS and 52 controls were examined for the associations between PCOS, body dissatisfaction, and eating attitudes. The results revealed that both hirsutism and high BMI in PCOS can contribute to body dissatisfaction and EDs. In addition, another general factor shown to contribute to EDs was the socio-cultural internalization of the perceived ideal thin body figure, which has lowered body dissatisfaction and self-esteem in the general population. However, there was no significant difference in BMI, socio-cultural internalization of ideal appearance, body dissatisfaction, and eating attitudes between PCOS and non-PCOS groups [36].

### 2.4. The Vicious Cycle of PCOS, Eating Disorders, and Obesity

Since hyperandrogenism and hyperinsulinemia in PCOS are two interrelated factors contributing to weight gain and body dissatisfaction, leading to the high prevalence of EDs, it is essential to explore their relationship and potential interventions at any step in this chain of events. Furthermore, the identification and assessment of EDs and eating behaviors might improve the treatment response to new anti-obesity medications resulting in significant improvement in the general health of women with PCOS.

Multiple potential mechanisms could explain how obesity can play a significant role in PCOS development and clinical presentation. In adolescence, the nutritional status remains a crucial indicator for puberty and menarche induction. A “critical body weight” is necessary to trigger the onset of puberty [37,38,39,40]. Leptin, a hormone that provides indirect information about an organism’s nutritional and metabolic status to the hypothalamic GnRH neuronal system, appears to regulate GnRH function indirectly through forebrain neurons [41]. Signalization of forebrain Kiss 1 neurons by leptin, which is elevated in children with obesity, acts as a permissive factor in the initiation and progression of puberty [42]. Increased hypothalamic ceramide content, which enhances paraventricular nucleus expression of SPTLC1 (a key enzyme for ceramide synthesis) and sympathetic ovarian innervations seem to be the responsible neuroendocrine mechanisms for the early onset of puberty in obese children [43]. Early puberty and menarche can result in adverse mental health and psychosocial consequences. Additional endocrine mechanisms for early puberty in obese girls include increased extragonadal aromatization of androgens to estrogen in adipose tissue [44]. Therefore, a larger area of aromatization can result in breast development and early thelarche. This premature activation of the GnRH axis seen in obesity is highly associated with hyperandrogenism and increased ovarian volume, two characteristic features of PCOS [45].

Obesity can also cause higher rates of irregular menses, amenorrhea, abnormal uterine bleeding, dysmenorrhea, premenstrual disorders, greater risk for infertility, pregnancy complications, breast and endometrial cancers, and PCOS. The most known mechanisms which explain those consequences are IR, high androgen levels, and low sex-hormone-binding globulin [46].

**Figure 1:**
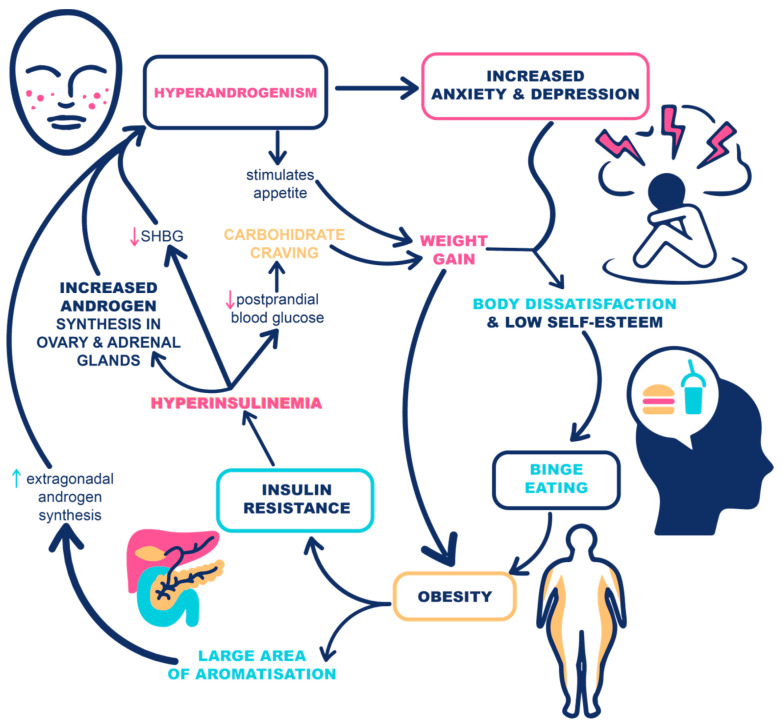
The potential mechanisms linking hyperandrogenism and insulin resistance with eating disorders in women with PCOS and obesity.

IR, the critical hormonal disturbance in obesity and metabolic syndrome, has a significant impact on the development and progression of PCOS and its clinical manifestations. In addition to IR related to obesity, PCOS is also related to IR that is intrinsically associated with the syndrome. Current evidence suggests that up to 75% of women with PCOS have impaired insulin action and that the degree of IR is often disproportionate to the BMI [47,48]. IR leads to hyperinsulinemia, which directly increases the bioavailability of sex steroids by stimulating the production of androgens by ovaries and adrenal glands and reduces sex-hormone-binding globulin levels (SHBG). In addition, the aromatase activity is also enhanced by hyperinsulinemia [49]. The potential mechanisms linking hyperandrogenism and hyperinsulinemia with EDs are summarized in Figure 1.

Based on the fact that obesity is a common finding in PCOS and aggravates many of its reproductive and metabolic features [50], a greater focus should be placed on more efficient treatment of obesity and, therefore, potentially stopping this vicious circle with one intervention at multiple steps. The anti-obesity treatment strategy should involve the identification of barriers to weight loss, with mental health being identified as one of the key predictors of the likelihood of successful weight loss intervention.

## 3. Eating Behavior

### 3.1. Regulation of Eating Behavior

Obesity is a disease in which the disbalance between energy intake and consumption leads to abnormally distributed and excess body fat that has direct and indirect consequences on whole-body health. Therefore, it is essential to carefully consider eating behavior that causes overeating along with insufficient energy expenditure. Eating behavior is a complex interplay of physiological, psychological, social, and genetic factors that influence meal timing, the quantity of food intake, and food preferences [51]. The brain controls it on three levels. Homeostatic eating is eating based on hunger and is mainly under hypothalamic control. It is a balance between feelings of satiety and hunger. Satiety is mediated by glucagon-like peptide 1 (GLP-1), peptide YY, oxyntomodulin, pancreatic polypeptide, and amylin, and hunger is mediated by ghrelin. Hedonic eating is eating based on pleasure and is mainly under mesolimbic control, mediated by feelings of wanting or liking to eat. A “want” to eat is mediated by dopamine, a hormone involved in the reward-behavior system. “Liking” to eat is associated with pleasure derived from eating and is mediated through opioid and cannabinoid receptors. Finally, executive function is the decision to eat, mediated primarily by the prefrontal cortex. Those brain regions are the site of action for behavioral interventions [18,52,53,54,55].

Neuroendocrine regulation between the brain, gut, and adipose tissue could be affected by PCOS. Insulin displays an inhibitory action on brain reactivity to food cues, but this effect is compromised by IR, frequently found in the PCOS population. Leptin, which was reported higher in women with PCOS [56], seems to have a controversial role in food intake regulation. It conveys an afferent signal to the central nervous system on body fat status, and in individuals with normal weight, it suppresses appetite and promotes energy expenditure. In obese patients, leptin is incapable of decreasing food intake and body weight due to leptin resistance [57]. Insulin and estradiol as stimulants and androgens as inhibitors of leptin production were demonstrated as hormonal regulators of leptin levels in some studies [58,59]. However, well-designed studies failed to find a significant correlation between serum leptin levels and Homeostasis Model Assessment (HOMA) or the degree of hyperinsulinemia in women with PCOS after adjusting for BMI [60], and even the results from interventional studies were controversial. In earlier studies, metformin decreased leptin levels both in obese and lean patients with PCOS [61], while, more recently, the studies did not confirm this evidence, suggesting that the effect of metformin on leptin levels could be mediated by its impact on body weight [62]. More data are necessary to explain the role of leptin in PCOS and its effect on eating behavior. The action of ghrelin, a potent orexigenic peripheral peptide, and neuropeptide Y, a potent orexigenic hypothalamic peptide, are deranged partially by the influence of insulin [63]. That might explain why excessive food intake due to affected neuroendocrine transmitters might account for the high rate of obesity in this population.

Besides mechanisms that try to explain how neuroendocrine regulation can lead to obesity in the PCOS population, another clinically useful concept is the characterization of obesity phenotype based on pathophysiological classification and eating behaviors. There are three measurable components that have an impact on eating behavior: quantity of food in every meal, time of different activities during the day, and specific content of the meal [51]. Their evaluation enables a clearer picture and suggests the potential individual intervention targeting one component in specific eating behavior.

### 3.2. Assessment of Eating Behavior

A study by Acosta et al. included 450 obese participants and performed different validated tests after an 8-h fasting period. They stratified obesity into four phenotypes based on eating behavior and energy expenditure: (1) Homeostatic eating behavior, which includes hungry brain (abnormal satiation), (2) hungry gut (abnormal satiety), (3) hedonic eating behavior, which is emotional eating, and (4) low energy expenditure (resting energy expenditure, non-exercise physical activity and thermogenic effect of food and exercise). Available methods which were performed to reveal those phenotypes were: nutrient drink test for satiation test; gastric emptying by scintigraphy for satiety test; validated questionnaires for emotional hunger; and indirect calorimetry for energy expenditure. Hedonic and homeostatic eating behaviors are two nutritional behaviors that tilt the energy balance towards increased intake [64]. This classification can be useful in the selection of personalized treatment for obesity, which is a very common feature of PCOS.

Eating chronotypes, referring to the time of different activities during the day, seem to be another key component in determining the risk of developing obesity. A cross-sectional study of 112 women with PCOS assessed participants’ chronotypes and their risk for obesity. Evening chronotypes showed significantly higher percentages of grade I and II obesity; most of them were smokers and exercised less regularly compared to those with neither or morning chronotypes [65]. Evening and late meals have also been more prevalent in PCOS women than controls in the study by Eleftheriadou et al. [66]. Furthermore, a recent study by Kulshreshtha et al. revealed that PCOS women differed significantly from non-PCOS women of the same weight in the timing of their breakfast and lunch intake rather than macronutrient distribution or caloric intake. Delayed breakfast and lunch were associated with PCOS independent of BMI, and late/missed breakfast was present in 40% of PCOS women [67]. These data suggest that chronotype assessment could be an effective tool to screen and change eating habits and general lifestyle in women with PCOS.

Assessment of eating content has also been recognized as an important determinant of eating behavior. Women with PCOS have low consumption of olive oil, legumes, fish, and nuts compared to healthy women, but they consume higher levels of simple carbohydrates, saturated fatty acids, and total fats. Women with PCOS also consume fewer complex carbohydrates, fibers, and monounsaturated fatty acids, which makes their eating content imbalanced [66]. Based on that evidence, diet and nutritional habits are very useful tools for PCOS management. Another relevant issue to take into consideration is the phenomenon of energy intake underreporting. This phenomenon has been studied by Giuseppe et al. in a pilot study. The study concluded that women with PCOS underreport foods rich in simple sugars rather than underreport their total dietary intake [68]. These results may have implications for the interpretation of diet and health correlations in this population since simple sugars are very involved in the weight gain process.

**Figure 2:**
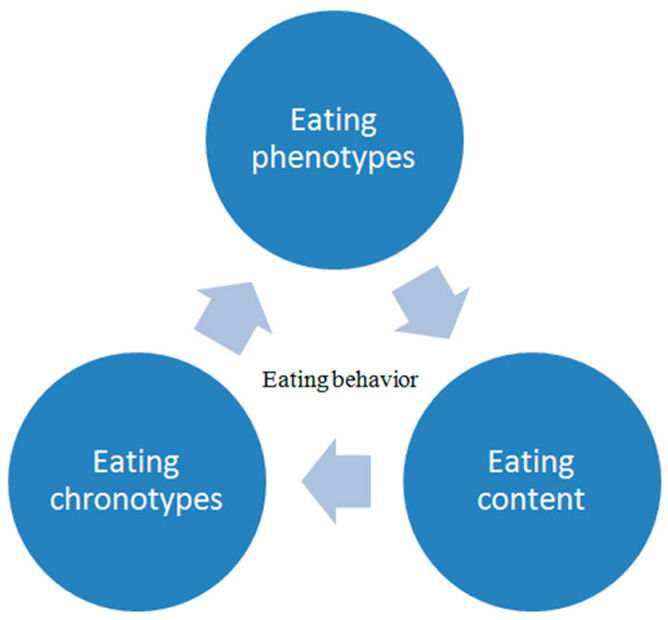
The process of assessment of eating behavior.

Altogether, the risk assessment process for eating behavior associated with weight gain and obesity is illustrated in Figure 2.

Moreover, the taste function as a component of eating behavior in PCOS women has been addressed by some authors. Cetik et al. assessed gustatory function by taste strips (sweet, sour, salty, bitter) and three different questionnaires. PCOS group had a lower sour and salty taste, whereas sweet and bitter taste scores were similar. They also concluded that hyperandrogenism was associated with lower total taste strip test score; however, the use of combined oral contraceptives did not alter taste function [69]. In preclinical studies, it was demonstrated that GLP-1 is locally synthesized in taste bud cells and its receptors exist on the gustatory nerves in close proximity to GLP-1-containing taste bud cells. This local paracrine GLP-1 signalizing seems to be involved in the perception of sweetness. Based on this finding, Jensterle et al. designed a randomized, single-blinded, placebo-controlled clinical trial in 30 women with obesity and PCOS to investigate if semaglutide modulates the perception of sweet taste in this population. This study may identify tongue and taste perception as a novel target for GLP-1 receptor agonists (GLP-1RAs) [70].

## 4. Assessment of Eating Disorders and Behavior to Improve Anti-Obesity Treatment in PCOS

Strong interrelation between obesity and PCOS, as well as the significant metabolic and reproductive improvements following weight loss intervention, showcase the importance of targeting excess weight in PCOS patients [71]. Currently, weight reduction presents the most efficient treatment for overweight and obese women with PCOS [72]. Different weight loss approaches are available, including diet and exercise, anti-obesity medications, and bariatric surgery when indicated. Variation in treatment responses directs the research work in finding personalized treatment programs for overweight and obese women with PCOS [73]. As with weight loss management in general, there is a lack of specific clinical parameters or phenotypes that could serve as accurate predictors for post-intervention weight loss response. The data for PCOS are scarce; therefore, we need to implement the current knowledge from the general population with obesity [64].

One important aspect of lifestyle intervention is diet. Caloric restriction has been shown to promote sporadic ovulation in obese women with PCOS, and improvements that go beyond reproductive outcomes have been demonstrated to occur in a wide range of patients [74]. In addition to caloric restriction, the content of eating should also be carefully addressed. Besides knowing its importance across various conditions, the Mediterranean diet has already been shown to have benefits in preventing metabolically unhealthy obesity due to its anti-inflammatory effects, its decrease in weight gain, and cell stability in PCOS women [75,76]. Furthermore, women with lean PCOS phenotype must be encouraged to consume a healthier diet in its composition, increasing the consumption of vegetables and fruit daily to ensure they have an adequate supply of various minerals, vitamins, and nutrients based mainly on the fact that there is no need to lose weight, just to maintain their weight with necessary micronutrients [77]. The second pillar of successful lifestyle intervention is exercise. A meta-analysis found that improvements in health outcomes are more dependent on exercise intensity than dose, supporting significant improvements in cardio-respiratory fitness, IR, and body composition from vigorous-intensity exercise. The minimum recommended aerobic activity is 120 min per week [78]. Achievable goals with lifestyle intervention, such as 5–10% weight loss in women with PCOS with excess weight, yield significant clinical improvements [28].

On the other end of potential weight loss interventions is bariatric surgery. It possesses a strategic therapeutical position in patients with obesity, and most international guidelines indicate its use for BMI over 40 or over 35 with at least one or more obesity-related co-morbidities [79]. According to the latest international evidence-based guidelines for the assessment and management of PCOS, bariatric surgery is generally considered second-line to improve fertility outcomes in PCOS with anovulation and BMI over 35 resistant to intensive lifestyle modification and/or pharmacotherapy [28]. A nonrandomized 12 month-follow up trial including 90 women with PCOS and BMI more or equal to 27.5 kg/m^2^ compared treatment with metformin, combined oral contraceptive, and cyproterone acetate within the first 6 months followed by 6 months of intervention with metformin alone or bariatric surgery. The study demonstrated that complete remission of PCOS in patients with obesity depends on the final BMI after weight loss, which again emphasizes the role of obesity treatment in this population and the beneficial effects of bariatric surgery in certain situations [80]. In addition, there is evidence that bariatric surgery can also change eating behaviors. Patients describe lower hunger levels, more postprandial fullness, lower portions, and lower calorie intake, which leads to significant negative energy balance and weight loss. In a one-year period, they achieve their energy balance, but the weight does not go back to baseline levels because it seems to be protected by a new set-point level [81].

The treatment gap between lifestyle intervention and bariatric surgery could be filled with the first and second generations of anti-obesity medications. Lately, the pharmacotherapy options for weight management have expanded with powerful tools that enable the use of prespecified weight loss amount as a primary biomarker and treatment outcome and achieve outcomes that are approaching the effects of bariatric surgery [82]. Currently, the approved medications to treat obesity are orlistat, phentermine–topiramate, bupropion–naltrexone, liraglutide, and semaglutide [83,84,85,86]. Studies that report their effects on weight loss and different clinical parameters are also available for the PCOS population.

Kumar et al. compared the effects of exercise, metformin, and orlistat on anthropometric parameters, lipid profile, endocrine parameters, and ovulation in PCOS. They concluded that orlistat is as effective as metformin in reducing weight and achieves similar ovulation rates in obese PCOS patients. However, orlistat has minimal side effects, is better tolerated compared with metformin, and also achieves significant improvements in lipid profile [87]. Another 24-week single-blinded study by Hirsch et al. compared the effects of sodium-glucose cotransporter-2 (SGLT2) inhibitors, phentermine/topiramate, exenatide, and metformin in different parameters, including weight, blood pressure, body composition, mean blood glucose, insulin sensitivity, and secretion in PCOS women and revealed that dual therapy with exenatide 2 mg weekly and dapagliflozine 10 mg daily was superior in terms of clinical and metabolic benefits in this population [88]. As for naltrexone/bupropion, we only found data on its positive outcome in depression in PCOS, which strongly correlates with EDs and obesity [89].

On the other hand, the amount of data evaluating the efficacy of GLP-1RAs in PCOS has been increasing over the last decade. GLP-1RAs have been investigated in overweight/obese women with PCOS in several single-center randomized control trials with considerable variation in the dosing regimen, follow-up duration, and outcome measurements. Most trials reported superior weight loss effects of GLP-1RAs compared to lifestyle changes or metformin, with additional metabolic, reproductive, and cardiovascular benefits in this population. However, their use is currently not widely accepted by the clinical community for treating this population, and the major concern is how to balance the reproductive and metabolic treatment strategies since the use of GLP-1RAs requires effective contraception while on therapy and a washout period before pregnancy [71].

A series of studies provide insights on GLP-1-induced modulation of food preference, eating behavior, and central nervous system response to visual and gustatory food cues as assessed by functional MRI [90,91,92,93]. It was demonstrated that endogenous GLP-1 and administration of GLP-1RAs affect central nervous system activation in response to viewing pictures of food items [91,92], which is related to the predictive value of food consumption and craving for food. Responsiveness to the actual palatable food consumption demonstrated that GLP-1 increase central nervous system response to the ingestion of palatable foods [90,93]. In individuals with type 2 diabetes, liraglutide decreased central nervous system activation after the short-term intervention, suggesting that these effects of GLP-1RAs on the central nervous system may contribute to the induction of weight loss [93]. Another study indicated that emotional eaters have altered brain responses to food cues and are less sensitive to the central effects of GLP-1 receptor activation with the acute infusion of exenatide [94]. In addition, the role of semaglutide on taste perception, along with a neural response to visual food cues in reward processing regions, is currently investigated in 30 women with obesity and PCOS [70].

With an increase in the number and efficacy of weight management tools, careful assessment of the clinical context is needed to enable personalized obesity treatment that yields the best outcomes for a specific patient. Knowing a patient’s individual circumstances, precise clinical sub-phenotyping, and regular monitoring are crucial components for the safe and effective use of the new tools. However, the field of personalized weight loss management in PCOS is mostly unexplored, and new trials will be needed to enhance our understanding of the direct impact that current and future weight management tools have on specific, currently unidentified PCOS phenotypes.

To overcome this gap in our knowledge and help guide clinicians and researchers throughout this period, particular emphasis will need to be placed on the trials of obesity treatment in general and combine their data with PCOS phenotypes based on Rotterdam criteria. For example, Acosta et al. assigned obese patients in different phenotypes based on their pathophysiologic differences and compared phenotype-guided treatment with non-phenotype guided treatment as below: abnormal satiation (hungry brain)—phentermine–topiramate extended-release or lorcaserin (which is now exempt from FDA approval), abnormal hedonic eating (emotional hunger)—naltrexone/bupropion extended release, abnormal satiety (hungry gut)—liraglutide, and low predicted energy expenditure (slow burn)—phentermine plus increased resistance training. In this pragmatic clinical trial, the phenotype-guided approach was associated with a statistically significant 1.75-fold greater weight loss after 12 months, with a mean weight loss of 15.9% compared with 9.0% in the non-phenotype-guided group. In addition, the proportion of patients who lost more than 10% of their weight in 12-month period was 79% in the phenotype-guided group compared to 34% in the non-phenotype-guided group [64]. Moreover, in the Canadian adult obesity clinical practice guideline, obtaining a comprehensive history to identify the root causes of weight gain, as well as physical, mental, and psychosocial barriers, is recommended, and the authors strongly emphasize that treating the root causes of weight gain is the foundation of obesity management and needs to take into account 4Ms: mechanical, metabolic, mental, and social milieu [95].

## 5. Conclusions

In this narrative review, we comprehensively covered the prevalence and underlying mechanisms behind common EDs and eating behaviors in PCOS and their implications for the management of women with PCOS.

The essential role of the frequent coexistence of mental disorders and PCOS is being increasingly recognized in the management of PCOS patients and influences the success of weight loss interventions. Hyperandrogenism, reproductive and metabolic disturbances, and the high prevalence of obesity contribute to body dissatisfaction as well as other psychological aspects. Patients frequently experience an extreme manifestation of disrupted eating behaviors, evidenced by the high prevalence of EDs in this population. Therefore, assessment and potential modification of EDs and eating-related behavior might be especially relevant to improve obesity treatment outcomes in this population. With an increase in the number and efficacy of weight management tools, careful assessment of the clinical context is needed to enable personalized obesity treatment that yields the best outcomes for a specific patient. Assessments of eating phenotypes, eating chronotypes, and eating content have been recognized as important determinants of eating behavior that could add to the personalization of the obesity treatment in PCOS and improve the final outcomes.

However, our review has some limitations that should be considered in the interpretation of the main conclusions. In the investigation of the prevalence of EDs in women with PCOS, there is a clear lack of unifying questionnaires or interviews to determine the specific prevalence in those studies. In addition, most of the included studies are relatively small and without proper designs to avoid selection bias. That could be a reason for the variation in the reported prevalence and possibly the potential overreporting of EDs in some studies. In addition, the data on eating behavior in PCOS are scarce. Larger and well-design studies are needed for the successful implementation of eating behavior assessment as a tool for personalized obesity treatment in PCOS.

## Figures and Tables

**Table 1:** Studies reporting the prevalence of eating disorders in PCOS.

Reference	Population Studied	Study Type	Eating Disorders	Prevalence in PCOS	Prevalence in Control Group	Questionnaire/ Interview	Other Remarks
[15]	103 women with PCOS, 103 controls	Cohort Study	Binge eating disorder	12.6% §	1.9%	Primary Care Evaluation of Mental Disorders (PRIME-MD PHQ) and the Beck Depression Inventory	Risk of depressive disorders, in general, was significantly higher in PCOS group; 30.5% of depressed women had binge eating disorder.
[18]	44 women with untreated PCOS	Cross-sectional study	Binge eating disorder	6.8%	-	Structured Clinical Interview (SCID-I) for the Diagnostic and Statistical Manual of Mental Disorders, 4th Edition (DSM-IV)	A total of 50% had psychiatric disorders, 33% major depression, and 13.6% generalized anxiety disorder.
			Bulimia nervosa	2.3%			
			Anorexia nervosa	0%			
[19]	60 women with PCOS	Prospective longitudinal study	Binge eating disorder	25% in PCOS group with depression (n = 24) and 22% in PCOS without depression (n = 36) ƛ	-	Primary Care Evaluation of Mental Disorders Patient Health Questionnaire (PRIME-MD PHQ), Beck Depression Inventory–II (BDI), Beck Anxiety Inventory (BAI)	Overall prevalence of depression was 40%.
[20]	455 women with PCOS	Cross-sectional study	Binge eating behavior	67%	-	Bulimia Investigatory test, Edinburgh (BITE), Food Cravings Questionnaire-Trait (FCQ-T), Three-Factor Eating Questionnaire-Revised 18 (TFEQ-18)	In total, 99% of PCOS patients reported food cravings.
			High-degree binge eating behavior	39%			
			Eating disorder	2%			
[21]	116 women with PCOS, 378 controls	Cross-sectional study	Binge eating disorder	93.1% §	82.5%	Arabic version of DASS-21 with 4 sections: demographics information, eating behaviors in past 2 weeks, depression subscale, health-related quality of life.	Risk of depression in PCOS was higher than in controls (*p* = 0.000).
							Health-related quality of life was impaired in PCOS group.
[22]	95 women with PCOS	Cross-sectional study	Bulimia nervosa	5.3%	-	Eating Attitudes Test (EAT); Eating Behavior Severity Scale (EBSS)	
			Subclinical anorexia nervosa	1.1%			
			Subclinical bulimia nervosa	10.5%			
[10]	148 women with PCOS, 106 controls	Cross-sectional study	All eating disorders	28.38% ƛ	18.87%	Eating Disorder Examination-Questionnaire (EDE-Q), Night Eating Questionnaire (NEQ), Hospital Anxiety and Depression Scale, and Health-Related Quality of Life Questionnaire (PCOSQ).	Elevated scores were noted for body shape and weight concern, anxiety, and depression in PCOS group.
			Anorexia nervosa	0% ƛ	0%		
			Bulimia nervosa	6.1% ƛ	5.66%		
			Binge eating disorder	17.6% ƛ	10.38%		
			Night eating syndrome	12.9% ƛ	12.38%		
[17]	875 women with PCOS, 7592 controls	Community-based cohort study	All eating disorders	11% §	7.6%	Rosenberg Self-Esteem Scale, Kessler psychological distress scale.	Obesity was associated with increased odds of low self-esteem and moderate to severe psychological distress.
			Anorexia nervosa	3.5% ƛ	3.4%		
			Bulimia nervosa	3.4% ƛ	2.6%		Underweight women had increased odds of eating disorders (not specified), low self-esteem, and psychological distress.
			others	6.4% §	3.4%		

§—The results were statistically significant (*p* ≤ 0.05). ƛ—The results were not statistically significant (*p* > 0.05).

## Data Availability

Not applicable.
